# Systematic Review with Network Meta-Analysis: Comparative Efficacy of Biologics in the Treatment of Moderately to Severely Active Ulcerative Colitis

**DOI:** 10.1371/journal.pone.0165435

**Published:** 2016-10-24

**Authors:** Adrian D. Vickers, Claire Ainsworth, Reema Mody, Annika Bergman, Caroline S. Ling, Jasmina Medjedovic, Michael Smyth

**Affiliations:** 1 RTI Health Solutions, Manchester, United Kingdom; 2 Takeda Development Center Americas, Inc., Deerfield, Illinois, United States of America; 3 Takeda Pharmaceutical GmbH, Zurich, Switzerland; 4 Takeda Development Centre Europe Ltd., London, United Kingdom; University Hospital Llandough, UNITED KINGDOM

## Abstract

**Background:**

Biological therapies are increasingly used to treat ulcerative colitis (UC).

**Aim:**

To compare the efficacy of biologics in adults with moderately-to-severely active UC, stratified by prior exposure to anti-tumour necrosis factor (anti-TNF) therapy.

**Methods:**

A systematic literature review was undertaken to identify studies of biologics approved for UC. Network meta-analysis was conducted for endpoints at induction and maintenance.

**Results:**

Seven studies were included in the meta-analysis of induction treatment for anti-TNF therapy-naïve patients. All biologics were more effective than placebo in inducing clinical response, clinical remission, and mucosal healing. Infliximab demonstrated a statistically significant improvement over adalimumab in clinical response (odds ratio [OR] [95% credible interval (CrI)]: 2.19 [1.35–3.55]), clinical remission (OR [95% CrI]: 2.81 [1.49–5.49]), and mucosal healing (OR [95% CrI]: 2.23 [1.21–4.14]); there were no other significant differences between biologics for induction efficacy. Five studies were included in the meta-analysis of maintenance treatment, two studies rerandomised responder patients at end of induction, and three followed the same patients ‘straight through’. To account for design differences, the number of responders at end of induction was assumed to be equivalent to the number rerandomised. Vedolizumab showed significantly different durable clinical response from comparators (OR [95% CrI] infliximab 3.18 [1.14–9.20], golimumab 2.33 [1.04–5.41], and adalimumab 3.96 [1.67–9.84]). In anti-TNF therapy-experienced patients, only vedolizumab and adalimumab could be compared. At induction, no significant differences in efficacy were seen. During maintenance, vedolizumab showed significantly improved rates of mucosal healing versus adalimumab (OR [95% CrI]: 6.72 [1.36–41.0]).

**Conclusions:**

This study expands the understanding of comparative efficacies of biologic treatments for UC, encompassing outcomes and populations not previously studied. All biologic treatments were effective for UC during induction. Vedolizumab demonstrated possible clinical benefits in the maintenance setting versus all comparators, irrespective of prior anti-TNF exposure and after adjusting for differences in study design.

## Introduction

Ulcerative colitis (UC) is characterised as a chronic condition in which the colon and rectum become inflamed and ulcerated [1, 2]. It has an incidence in the United Kingdom of approximately 10 per 100,000 people annually and prevalence of approximately 240 per 100,000 [3]. It has recently been estimated that approximately 593,000 people in the United States have UC [4]. The symptoms of UC can lead to a substantial negative impact on patient quality of life [5] and incur a significant economic burden, including both direct medical costs and indirect costs associated with absenteeism and productivity loss [6].

Current treatment strategies for UC are not curative; even surgery may be followed by ongoing morbidity [7]. Rather, pharmacological therapies are used to treat acute, active disease and to maintain response and prevent relapse among patients in remission [7]. Treatment for mild to moderate UC consists of aminosalicylates, steroids and immunosuppressants [8]. In the last decade, biological therapies have been approved for the treatment of moderately to severely active UC. Initially, these were all anti-cytokine agents; adalimumab, golimumab and infliximab all suppress the immune system by blocking the activity of tumour necrosis factor (anti-TNFs) [9–11]. More recently, vedolizumab, which is an integrin receptor antagonist that results in gut-selective anti-inflammatory activity, has been introduced [12]. A recent Cochrane systematic review and meta-analysis concluded that vedolizumab is superior to placebo as an induction and maintenance therapy for UC [13]. Clinical remission with biological therapies has been demonstrated in patients who inadequately respond to conventional medications, and evidence suggests that withdrawal from corticosteroids may be possible for some patients [14].

There are no published, head-to-head randomised controlled trials (RCTs) comparing the efficacy and safety of the different biologics for the treatment of moderately to severely active UC. Although some indirect comparisons assessing the efficacy of different biologics have been published for UC [15–17], no indirect comparisons have been conducted to assess the comparative efficacy of approved biologics (adalimumab, infliximab, golimumab, and vedolizumab) for the treatment of moderately to severely active UC in patients with prior exposure to anti-TNF–therapy. This study builds upon previous analyses conducted in the anti-TNF–naïve subpopulation [15] by including additional data for mucosal healing for vedolizumab and attempting to make a comparison of all the approved biologics in the network of evidence in the maintenance setting. Performing analyses by prior exposure to anti-TNF therapy captures the clinical differences between these subpopulations [18, 19] and recognises that, when assessing the relative efficacy of biologics, it may not be appropriate to pool patients for whom prior anti-TNF therapy was unsuccessful with those who are naïve to anti-TNF therapy at initiation [20, 21].

Furthermore, no indirect comparisons have been conducted in the maintenance setting stratified by prior exposure to anti-TNF therapy. This is partly owing to the differing study designs seen in the maintenance setting [15]; the studies of vedolizumab and golimumab rerandomised patients responding at the end of the induction phase to maintenance therapy. This rerandomisation is intended to reflect real clinical practice in which a patient would be assessed at the end of induction and subsequent treatment decisions would be made based on response; it emulates recent guidance on study design by regulatory bodies on the separation of induction and maintenance phases [22]. In comparison, in the studies of adalimumab and infliximab, patients were randomised prior to induction therapy and remained on that treatment to the end of the maintenance phase.

This systematic review and meta-analysis aimed to evaluate the comparative efficacy and safety of approved biological therapies (adalimumab, infliximab, golimumab, and vedolizumab) for the treatment of moderately to severely active UC, stratified by prior exposure to anti-TNF therapy, in both the induction and maintenance setting.

## Materials and Methods

We conducted a systematic literature review in line with Cochrane methodology [23] and following Preferred Reporting Items for Systematic Reviews and Meta-Analyses recommendations [24] (see S1 File), according to the protocol developed in April 2013. An updated protocol was developed in February 2014 and was used to conduct an update of the systematic review. The meta-analysis was conducted according to the framework of Dias and colleagues [25].

### Data sources and searches

A systematic literature search was conducted in MEDLINE, Embase and the Cochrane library databases from initiation until 11 February 2014 by RTI-HS. The search included terms for UC, combined with the medicines of interest (vedolizumab, infliximab, adalimumab and golimumab). The search was limited to RCTs, systematic literature reviews, and meta-analyses and studies in humans. Language restrictions were not applied. The MEDLINE literature search strategy for the update of the systematic review is presented in S2 File.

We also searched the ClinicalTrials.gov database and the World Health Organization’s International Clinical Trials Registry Platform Search Portal for ongoing studies of the drugs of interest. Abstracts from Digestive Disease Week (2009–2013) and European Crohn’s and Colitis Organisation (2009–2013) were captured within the searches of Embase. Bibliographic reference lists of key systematic reviews and meta-analyses identified were reviewed for relevant publications.

### Study selection

The titles and abstracts of published articles identified were reviewed by two independent reviewers from the RTI-HS team to determine each study’s eligibility using prespecified criteria. The full texts of included studies were also obtained and reviewed by two investigators. Where consensus was not reached or if there was any uncertainty about the included studies, a third researcher was consulted.

Studies were eligible for inclusion if they were RCTs or prospective studies with more than one treatment arm and assessed the efficacy or safety of biological agents for the treatment of patients with UC. We considered only biological agents that had market authorisation or were anticipated to obtain a license within the next year for use in UC by either the United States Food and Drug Administration or the European Medicines Agency. RCTs were eligible for inclusion in the network regardless of country, phase (2 or 3), or source of support.

### Data extraction and quality assessment

All data were extracted by one reviewer and quality checked by an independent reviewer from RTI-HS. Any data discrepancies were discussed and resolved. Details on study design, treatment information and patient baseline characteristics were extracted along with the definition, time point and proportion of patients achieving each of the following efficacy outcome measures: clinical response, durable clinical response, clinical remission, durable clinical remission, Inflammatory Bowel Disease Questionnaire (IBDQ) response, steroid-free (SF) remission, mucosal healing and durable mucosal healing. Similarly, data were extracted on the definition, time point and proportion of patients experiencing each of the following safety outcomes measures: surgery required, hospitalisations, overall adverse events (AEs), serious AEs, discontinuations due to AEs, severe AEs and fatal AEs. For quality-of-life outcomes (IBDQ, SF-36 Health Survey), data were extracted on the time point, number of patients with a result, mean score and mean change in score from baseline.

We extracted data for subpopulations of patients who were naïve to, had prior exposure to, or had failed treatment with anti-TNF therapy. Different doses of the same treatment were considered as separate interventions. Data for outcome measures were extracted for each study at the end of induction and maintenance phase for each subgroup where available.

To assess the quality of the included studies, a risk of bias assessment was conducted by RTI-HS. The assessment as set out in the National Institute for Health and Care Excellence (NICE) “specification for manufacturers” [26] was applied to each study.

### Data synthesis and analysis

Six endpoints (response, remission, SF remission, mucosal healing, discontinuations due to AEs, serious AEs) were considered for meta-analysis because these were the most commonly reported, with similar definitions, in the studies identified. Each of the chosen endpoints was analysed separately at each relevant time point (induction and maintenance) and for each population (anti-TNF therapy-naïve subpopulation and anti-TNF therapy-experienced/failure subpopulation) where data were available. In total, 24 mixed-treatment comparison (MTC) analyses (not including sensitivity analyses) were conducted. Using this form of meta-analysis allows analysis of direct and indirect comparisons to produce estimates of effect (odds ratios [ORs] with 95% credible intervals [CrIs]) for all possible pairwise comparisons despite a lack of direct comparison in a head-to-head fashion in the included clinical studies. In this circumstance, the analysis has allowed indirect comparisons between biologics using placebo as a common comparator. This paper focuses on the analysis of the anti-TNF therapy-naïve and anti-TNF therapy-experienced/failure subpopulations in recognition of the clinical differences between them [18, 19]. In addition, data are only presented for labelled doses of the licensed biologics.

#### Time points for analysis

For induction analyses, the primary time point presented was used for all comparators. For maintenance analyses, data presented for 52 or 54 weeks were used in the analyses. Shorter duration studies (e.g., 24 or 26 weeks) were excluded because over this time period it was not possible to differentiate the effect of induction treatment from that of maintenance treatment.

#### Outcomes for analysis

**Clinical response** at the end of induction was defined as a reduction in complete Mayo score of ≥3 points and ≥30% change from baseline with an accompanying decrease in rectal bleeding subscore of ≥1 point or absolute rectal bleeding subscore of ≤1 point.

**Durable clinical response**, defined as clinical response at both start and end of maintenance, was used for treatment efficacy in a maintenance setting. In studies in which patients were rerandomised into maintenance treatment based on induction clinical response, durable response rates were presented and thus used in the analyses. For analysis of durable clinical response in those studies that did not rerandomise, the number of responders at the end of induction and at the end of maintenance was used as a proxy of durable response.

**Clinical remission** was defined as a complete Mayo score of ≤2 points and no individual subscore >1 point. Owing to lack of available data for durable clinical remission (i.e., remission at both end of induction and end of maintenance), clinical remission data were analysed separately at the end of induction and at the end of maintenance.

**Steroid-free (SF) remission** was defined as patients using oral corticosteroids at baseline who discontinued corticosteroids and were in clinical remission at the end of maintenance. Although SF remission data were available for anti-TNF therapy-naïve patients in Active Ulcerative Colitis Trial 1 (ACT 1) [27], patients in that study were not rerandomised at the end of induction. Thus, the number of responders at the end of induction who also received corticosteroids at the beginning of the study was not available, making a comparison with the anti-TNF therapy-naïve population in GEMINI-1 [28–35], in which patients were rerandomised, unfeasible. No other included studies reported data on SF remission by anti-TNF–therapy exposure subpopulation. Hence, this outcome has not been included in the analyses.

**Mucosal healing** was defined as a Mayo endoscopic subscore of ≤1 point. Mucosal healing data were analysed separately at the end of induction and at the end of maintenance.

#### Safety

The proportion of patients who discontinued treatment because of AEs also was examined. Where available, data for the intention-to-treat population were used to ensure that the population reflected that used for the clinical analyses. Other safety endpoints, including serious AEs, were not suitable for MTC owing to a lack of events and inconsistent reporting.

#### Subgroup analysis

Although all studies included patients who were anti-TNF therapy naïve, data for patients with prior anti-TNF–therapy exposure were available only for vedolizumab and adalimumab, and the definitions of these groups differed. In the vedolizumab studies, patients who failed previous anti-TNF therapy (defined as patients with inadequate response to, loss of response to or intolerance of anti-TNF therapy) were analysed. In comparison, the adalimumab studies reported results for anti-TNF therapy-experienced patients, including those patients who may have had a partial response or relapse following anti-TNF therapy. Our analyses used the anti-TNF therapy-failure population in the vedolizumab studies versus the anti-TNF therapy-experienced population in the adalimumab studies.

#### Analytic methods

All analyses were performed using a combination of R [36] and OpenBUGS [37]. Both fixed-effects and random-effects MTCs were conducted where closed loops or duplicate comparisons existed in a network. However, all of the networks were too small to give reliable results for the random-effects models. Therefore, only the results from the fixed-effects models have been reported.

The methods used to fit the Bayesian MTCs follow those of Lu and Ades [38] and Dias and colleagues [25]. The R package R2WinBUGS [39] was used to run OpenBUGS from within R. These models assumed binomial distributions and used a logistic link function. For all analyses conducted using OpenBUGS, the following model specifications were used: three chains, burn-in of 20,000 iterations, total of 60,000 iterations, thin rate of 50 and uninformative priors. Different runs with different priors showed that the choice of prior had negligible effect on the results. Checks for convergence and lack of autocorrelation were also performed. Owing to the small networks, convergence was easily achieved. There were no patterns in iteration plots and Gelman-Rubin diagnostics all gave 1.0.

The Bayesian results also were validated through the use of frequentist techniques described as follows. It was observed that the point estimates and CrIs from the Bayesian analyses matched very closely to those from the frequentist MTCs. The method used to conduct the frequentist MTCs was adapted from the method described by Lumley [40] and van der Valk [41] for a continuous endpoint. This approach was further extended to produce Bayesian-style predictions by generating simulations derived from the point estimates and variance-covariance matrix. This followed the methods described by Gelman and Hill [42] and Wood [43].

## Results

### Systematic literature review

**Fig 1:**
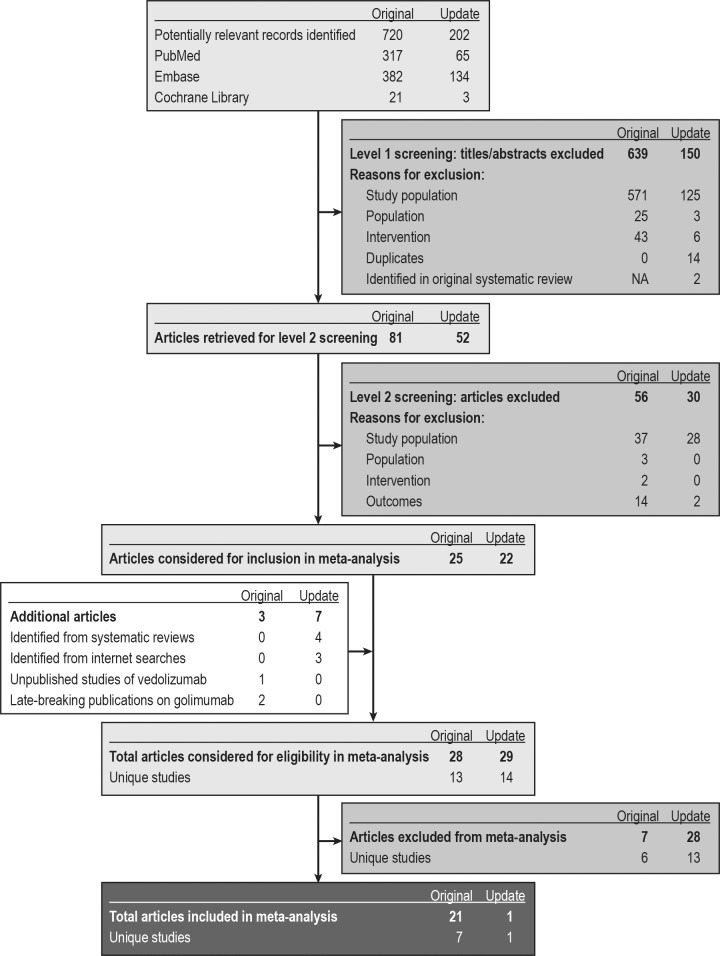
PRISMA diagram: identification and selection of sources. NA, not applicable; PRISMA, Preferred Reporting Items for Systematic Reviews and Meta-Analyses.

Fig 1 summarises the search and selection of evidence. We identified 22 publications reporting the results of eight studies: ACT 1 [27], ACT 2 [27], Ulcerative Colitis Long-Term Remission and Maintenance With Adalimumab 1 (ULTRA 1) [44], ULTRA 2 [45, 46], Program of Ulcerative Colitis Research Studies Utilizing an Investigational Treatment–Subcutaneous (PURSUIT-SC) [47], Program of Ulcerative Colitis Research Studies Utilizing an Investigational Treatment–Maintenance (PURSUIT-M) [48], GEMINI 1 [28–35], and NCT00853099 (Suzuki et al.) [49]. All eight studies were multicentre, randomised, double-blind, placebo-controlled trials investigating the efficacy and safety of four biological agents (adalimumab, infliximab, golimumab, and vedolizumab) as induction or maintenance therapy for adults with moderately to severely active UC. None of the eight studies were head-to-head comparisons of biological agents, so all results are based on indirect comparisons. No prospective non-RCTs with more than one treatment arm were identified for inclusion in the review.

In most studies, patients were randomly assigned once, at study outset, to receive induction therapy or matching placebo followed by maintenance therapy for a longer phase. However, in two studies (PURSUIT-M and GEMINI 1), only patients who achieved clinical response at induction were eligible and were rerandomised to placebo or active treatment for maintenance therapy.

**Fig 2:**
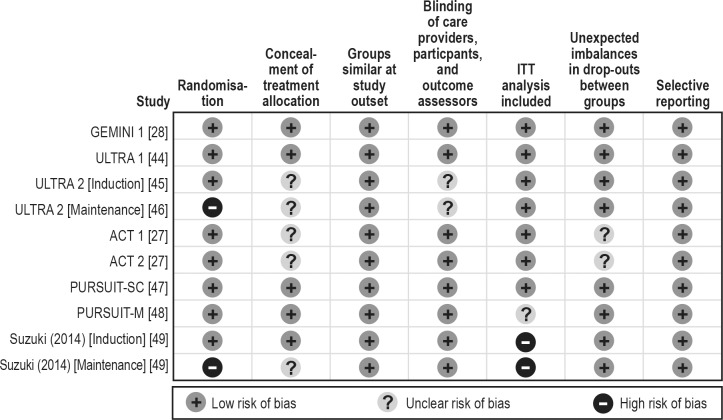
Risk of bias assessment of trials included in the mixed-treatment comparison. ITT, intent-to-treat.

Table 1 presents characteristics of the patients in the included studies. The mean age of patients ranged from 38 to 43 years, the mean disease duration ranged from 5 to 9 years, and 53% to 73% of patients were male. Six studies (ACT 1, ACT 2, ULTRA 1, NCT00853099, PURSUIT-SC, and PURSUIT-M), recruited only treatment-naïve patients, whereas in the other two studies (ULTRA 2 and GEMINI 1), randomisation had been stratified by prior exposure to anti-TNF therapies. For each biological agent, we present outcomes for the dose and administration approved in the respective summary of product characteristics for each agent. Table 2 presents a summary of the available data in the evidence base. Fig 2 presents a summary of quality assessment across the studies.

**Table 1:** Included trials/treatments and important patient characteristics.

Study name or code	Interventions	Number randomised	Week of analysis (primary time point)	Mean age (years)	% Male	% Naïve	Mean disease duration (years)
**Induction**							
GEMINI 1 [29]	PBO	149	6	41.2	62	51	7.1
	VDZ 300 mg IV at week 0 and 2 and every 8 weeks thereafter	225	6	40.1	59	58	6.1
ULTRA 1 [44]	PBO	130	8	38.4	63	100	5.4^a^
	ADA 160 mg SC at week 0, followed by 80 mg at week 2 and then 40 mg every other week	130	8	37.9	64	100	6.1^a^
ULTRA 2 [45, 46]	PBO	258	8	41.3	62	59	8.5
	ADA 160 mg SC at week 0, followed by 80 mg at week 2 and then 40 mg every other week	260	8	39.6	57	61	8.1
ACT 1 [27]	PBO	121	8	41.4	60	100	6.2
	IFX 5 mg/kg IV at week 0, followed by 5 mg/kg at 2 and 6 weeks and every 8 weeks thereafter	122	8	42.4	65	100	5.9
ACT 2 [27]	PBO	121	8	39.3	58	100	6.5
	IFX 5 mg/kg IV at week 0, followed by 5 mg/kg at 2 and 6 weeks and every 8 weeks thereafter	120	8	40.5	63	100	6.7
PURSUIT-SC [47]	PBO	331	6	39.0	53	100	6.0
	GLM 200 mg SC at week 0, followed by 100 mg at week 2 and then 100 mg every 4 weeks	331	6	40.0	54	100	6.4
Suzuki, 2014 [49]	PBO	96	8	41.3	72.9	100	7.8
	ADA 160 mg/80 mg	90	8	42.5	67.8	100	7.8
**Maintenance**							
GEMINI 1 [28, 29, 30, 32, 33, 34]	PBO	126	52	40.3	55	63	7.8
	VDZ 300 mg IV every 8 weeks	122	52	41.0	57	59	6.2
ULTRA 2 [45, 46]	PBO	246^b^	52	40.5	58	87	NR
	ADA 40 mg SC every other week	248^b^	52	40.5	58	87	NR
ACT 1 [27]	PBO	121^b^	54	41.4	60	100	6.2
	IFX 5 mg/kg IV every 8 weeks	122^b^	54	42.4	65	100	5.9
PURSUIT-M [48]	PBO	156	54	40.2	48	100	6.9
	GLM 100 mg every 4 weeks	154	54	39.1	58	100	7.2
Suzuki, 2014 [49]	PBO	96^b^	52	NR	NR	100	NR
	ADA 40 mg SC every other week	177^b^	52	NR	NR	100	NR

ADA, adalimumab; GLM, golimumab; IFX, infliximab; IV, intravenously; NR, not reported; PBO, placebo; SC, subcutaneous; VDZ, vedolizumab.

^a^ Median disease duration.

^b^ These studies were not rerandomised at maintenance, so the number of patients who responded at the end of induction has been used in this table.

**Table 2:** Treatments and endpoints available for meta-analysis.

Biologic studied	Trial	Population	Response	Remission	Discontinuation due to adverse event	Mucosal healing
**Induction**						
VDZ	GEMINI 1 [28, 30, 31, 32, 33, 34, 50]	Naïve subpopulation	VDZ: 69/130 (53%)^a^; PBO: 20/76 (26%)^a^	VDZ: 30/130 (23%); PBO: 5/76 (7%)	VDZ: 0/130 (0%); PBO: 3/76 (4%)	VDZ: 64/130 (49%); PBO: 19/76 (25%)
		Failure subpopulation	VDZ: 32/82 (39%)^a^; PBO: 13/63 (21%)^a^	VDZ: 8/82 (10%); PBO: 2/63 (3%)	VDZ: 0/82 (0%); PBO: 2/63 (3%)	VDZ: 25/82 (30%); PBO: 13/63 (21%)
ADA	ULTRA 1 [44]	Naïve (ITT)	ADA: 71/130 (55%)^a^; PBO: 67/130 (45%)^a^	ADA: 24/130 (18%); PBO: 12/130 (9%)	ADA: 5/130 (4%); PBO: 5/130 (4%)	ADA: 61/130 (47%); PBO: 54/130 (42%)
	ULTRA 2 [45, 46]	Naïve subpopulation	ADA: 89/150 (59%)^a^; PBO: 56/145 (39%)^a^	ADA: 32/150 (21%); PBO: 16/145 (11%)	NA	ADA: 74/150 (49%); PBO: 51/145 (35%)
		Experienced subpopulation	ADA: 36/98 (37%)^a^; PBO: 29/101 (29%)^a^	ADA: 9/98 (9%); PBO: 7/101 (7%)	NA	ADA: 28/98 (29%); PBO: 27/101 (27%)
	Suzuki, 2014 [49]	Naïve (full analysis set)	ADA: 45/90 (50%)^a^; PBO: 34/96 (35%)^a^	ADA: 9/90 (10%); PBO: 11/96 (11%)	ADA: 6/90 (7%); PBO: 4/96 (4%)	ADA: 37/90 (41%); PBO: 29/96 (30%)
IFX	ACT 1 [27]	Naïve (ITT)	IFX: 84/121 (69%)^a^; PBO: 45/121 (37%)^a^	IFX: 47/121 (39%); PBO: 18/121 (15%)	NA	NA
	ACT 2 [27]	Naïve (ITT)	IFX: 78/121 (64%)^a^; PBO: 36/123 (29%)^a^	IFX: 41/121 (34%); PBO: 7/123 (6%)	NA	IFX: 73/121 (60%); PBO: 38/123 (31%)
GLM	PURSUIT-SC [47]	Naïve (ITT)	GLM: 133/257 (52%)^a^; PBO: 76/256 (30%)^a^	GLM: 48/257 (19%); PBO: 16/256 (6%)	GLM: 1/331 (0%); PBO: 3/330 (1%)	GLM: 111/257 (43%); PBO: 73/256 (29%)
**Maintenance**						
VDZ	GEMINI 1 [28, 30, 32, 33, 34, 35, 50]	Naïve subpopulation	VDZ: 47/72 (65%)^b^; PBO: 21/79 (27%)^b^	VDZ: 33/72 (46%); PBO: 15/79 (19%)	VDZ: 3/79 (4%); PBO: 9/88 (10%)	VDZ: 43/72 (60%); PBO: 19/79 (24%)
		Failure subpopulation	VDZ: 20/43 (47%)^b^; PBO: 6/38 (16%)^b^	VDZ: 16/43 (37%); PBO: 2/38 (5%)	VDZ: 4/43 (9%); PBO: 6/38 (16%)	VDZ: 18/43 (42%); PBO: 3/38 (8%)
ADA	ULTRA 2 [45, 46]	Naïve subpopulation	ADA: 55/89^c^ (62%)^b^; PBO: 35/56^c^ (63%)^b^	ADA: 33/89^c^ (37%); PBO: 18/56^c^ (32%)	NA	ADA: 47/89^c^ (53%); PBO: 28/56^c^ (50%)
		Experienced subpopulation	ADA: 20/29^c^ (69%)^b^; PBO: 10/36^c^ (28%)^b^	ADA: 10/29^c^ (34%); PBO: 3/36^c^ (8%)	NA	ADA: 15/29^c^ (52%); PBO: 10/36^c^ (28%)
	Suzuki, 2014 [49]	Naïve (full analysis set)	ADA: 25/82^c^ (30%)^b^; PBO: 6/34^c^ (18%)^b^	ADA: 19/82^c^ (23%); PBO: 2/34^c^ (6%)	ADA: 22/177 (12%); PBO: 6/96 (6%)	ADA: 51/82^c^ (62%); PBO: 15/34^c^ (44%)
IFX	ACT 1 [27]	Naïve (ITT)	IFX: 55/84^c^ (65%)^b^; PBO: 24/45^c^ (53%)^b^	IFX: 42/84^c^ (50%); PBO: 20/45^c^ (44%)	IFX: 10/121 (8%); PBO: 11/121 (9%)	IFX: 55/84^c^ (65%); PBO: 22/45^c^ (49%)
GLM	PURSUIT-M [48]	Naïve (ITT)	GLM: 72/153 (47%)^b^; PBO: 49/156 (31%)^b^	GLM: 51/153 (33%); PBO: 35/156 (22%)	GLM: 8/154 (5%); PBO: 10/156 (6%)	NA^d^

ADA, adalimumab; GLM, golimumab; IFX, infliximab; ITT, intention-to-treat; PBO, placebo; VDZ, vedolizumab.

Note: “NA” indicates data were not available; data are presented for n/N (%) unless otherwise stated.

^a^ Clinical response at the end of induction.

^b^ Durable clinical response, defined as clinical response at both start and end of maintenance, was used for treatment efficacy in a maintenance setting.

^c^ Study was not rerandomised at the end of induction; the number of responders at the end of induction has been used as a proxy for total number of patients, to estimate the percentage of responders as the end of induction and the end of maintenance, i.e. as a proxy for durable response.

^d^ Although mucosal healing data were available for PURSUIT-M [48], they were for patients who achieved mucosal healing at both week 34 and week 50 and were not therefore comparable with data from the other studies.

### Efficacy and safety of biological agents in the anti-TNF therapy-naïve subpopulation

Eight randomised studies of biological agents versus placebo contributed to this analysis (7 induction, 5 maintenance; Table 2).

#### Induction

**Fig 3:**
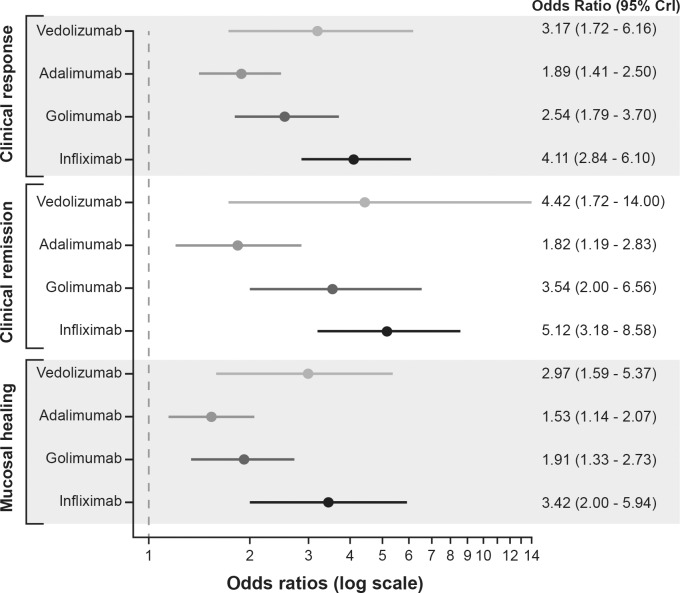
Forest plot of the odds ratios for biologics vs. placebo for anti-TNF therapy-naïve patients in induction studies. CrI, credible interval; TNF, tumour necrosis factor. Note: Adalimumab induction dose: 160 mg followed by 80 mg; vedolizumab induction dose: 300 mg; golimumab induction dose: 200 mg subcutaneous at week 0, followed by 100 mg at week 2 and then 100 mg every 4 weeks; infliximab induction dose: 5 mg/kg intravenously at week 0, followed by 5 mg/kg at 2 and 6 weeks.

**Fig 4:**
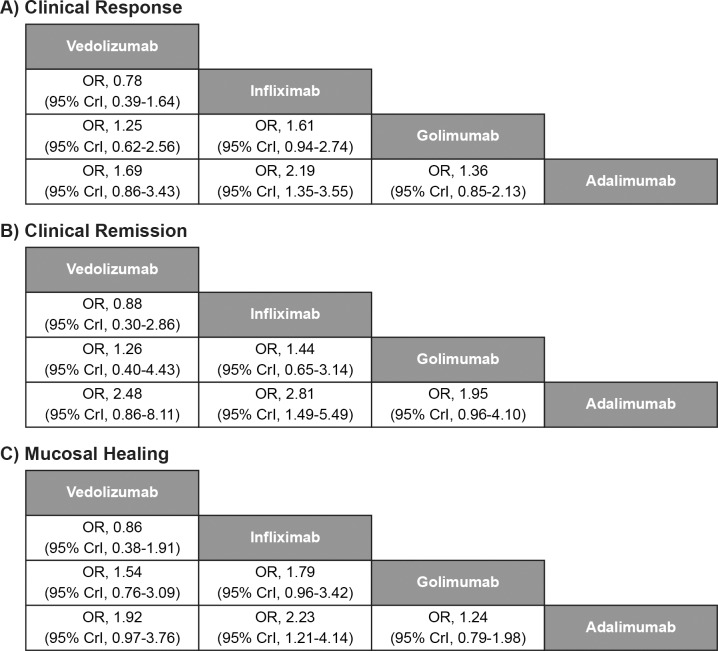
Comparative efficacy of biological agents as induction therapy for anti-TNF therapy-naïve subpopulation. CrI, credible interval; OR, odds ratio; TNF, tumour necrosis factor. Note: Treatment effect estimates come from Bayesian mixed-treatment comparison. ORs >1.0 favour the treatment in the left upper square. To obtain ORs for comparison in the opposite direction, reciprocals should be calculated.

All biologics (vedolizumab, adalimumab, golimumab and infliximab) showed significantly better clinical response, clinical remission and mucosal healing than placebo during the induction phase (Fig 3). Infliximab demonstrated a significant improvement over adalimumab in clinical response (OR [95% CrI], 2.19 [1.35–3.55]), clinical remission (OR [95% CrI], 2.81 [1.49–5.49]), and mucosal healing (OR [95% CrI], 2.23 [1.21–4.14]) at induction (Fig 4). There was no evidence to suggest differences between infliximab and vedolizumab, between infliximab and golimumab, or between labelled doses of the other licensed treatments (vedolizumab, adalimumab, and golimumab) for clinical response, clinical remission, or mucosal healing. Vedolizumab showed significantly better results for discontinuation due to AEs than adalimumab (0/130 patients vs. 11/220 patients, respectively, OR [95% CrI], 0.00 [0.00–0.19]); however, the results were from a smaller network of evidence.

#### Maintenance

Five randomised studies contributed to this analysis (Table 2). In two of the five maintenance studies (PURSUIT-M and GEMINI 1), only patients who achieved clinical response at induction were eligible and were rerandomised to placebo or active treatment for maintenance therapy. The maintenance analysis presented includes the ULTRA 2, ACT 1, and Suzuki and colleagues [49] studies, which did not rerandomise after induction.

**Fig 5:**
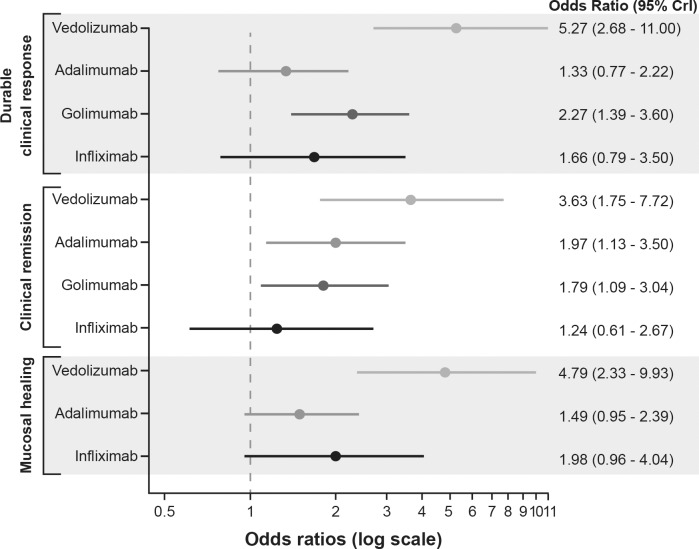
Forest plot of the odds ratios for biologics vs. placebo for anti-TNF therapy-naïve patients in maintenance studies. CrI, credible interval; TNF, tumour necrosis factor. Note: Adalimumab maintenance dose: 40 mg every other week; vedolizumab maintenance dose: 300 mg every 8 weeks; golimumab maintenance dose: 100 mg every 4 weeks; infliximab maintenance dose: 5 mg/kg intravenously every 8 weeks.

Vedolizumab and golimumab both showed significantly better durable clinical response than placebo during the maintenance phase (Fig 5). All biologics, except infliximab, showed significantly better clinical remission at maintenance than placebo. Only vedolizumab showed significantly better mucosal healing at maintenance than placebo (OR [95% CrI], 4.79 [2.33–9.93]).

**Fig 6:**
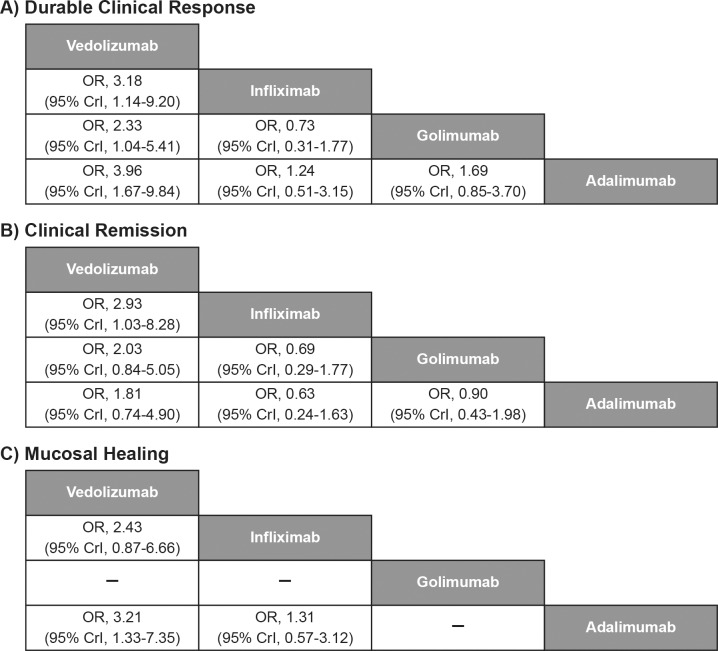
Comparative efficacy of biological agents as maintenance therapy for anti-TNF therapy-naïve subpopulation. CrI, credible interval; OR, odds ratio; TNF, tumour necrosis factor. Note: Treatment effect estimates come from Bayesian mixed-treatment comparison. ORs >1.0 favour the treatment in the left upper square. To obtain ORs for comparison in the opposite direction, reciprocals should be calculated.

Vedolizumab showed significantly better durable clinical response than adalimumab (OR [95% CrI], 3.96 [1.67–9.84]), infliximab (OR [95% CrI], 3.18 [1.14–9.20]), and golimumab (OR [95% CrI], 2.33 [1.04–5.41]) at maintenance (Fig 6). Vedolizumab also showed a significant improvement in clinical remission over infliximab (OR [95% CrI], 2.93 [1.03–8.28]) and significant improvement in mucosal healing over adalimumab (OR [95% CrI], 3.21 [1.33–7.35]) at maintenance. Vedolizumab (3/79 patients) showed significantly better results for discontinuation due to AEs than adalimumab (22/177 patients, OR [95% CrI], 0.14 [0.02–0.67]) and golimumab (14/154 patients, OR [95% CrI], 0.21 [0.03–0.99]).

### Efficacy and safety of biological agents in the anti-TNF therapy-experienced/failure subpopulation

Our analyses used the anti-TNF therapy-failure population in the vedolizumab study (GEMINI 1) versus the anti-TNF therapy-experienced population in the comparator study (ULTRA 2), and comparisons were conducted for the available outcomes of clinical response, durable clinical response, clinical remission and mucosal healing.

#### Induction

Vedolizumab showed significant improvement in clinical response over placebo (OR [95% CrI], 2.5 [1.2–5.5]); in other comparisons with placebo, significant differences were not seen (Table 3). There was no evidence to suggest differences between adalimumab and vedolizumab for clinical response, clinical remission, or mucosal healing (Table 3).

**Table 3:** Comparative efficacy of biological agents for induction and maintenance therapy for anti-TNF therapy-experienced subpopulation.

	Odds ratio (95% CrI)		
Time point (Endpoint)	Vedolizumab vs. adalimumab	Vedolizumab vs. placebo	Adalimumab vs. placebo
**Induction**			
Clinical response	1.74 (0.69–4.45)	2.51* (1.18–5.48)	1.43 (0.79–2.64)
Clinical remission	2.72 (0.43–23.79)	3.66 (0.87–27.98)	1.37 (0.47–4.03)
Mucosal healing	1.56 (0.57–4.22)	1.70 (0.80–3.81)	1.09 (0.60–2.10)
**Maintenance**			
Durable clinical response	2.04 (0.44–9.01)	4.89* (1.74–15.89)	2.47 (0.90–6.99)
Clinical remission	3.40 (0.40–32.52)	12.14* (3.14–78.38)	3.60* (1.01–18.23)
Mucosal healing	6.72* (1.36–41.17)	9.09* (2.74–40.06)	1.36 (0.50–3.91)

CrI, credible interval; OR, odds ratio; TNF, tumour necrosis factor.

* = significant.

#### Maintenance

Both vedolizumab and adalimumab were significantly better than placebo for clinical remission at maintenance (ORs [95% CrI], 12.0 [3.14–78.0] and 3.6 [1.01–18.0], respectively). However, only vedolizumab demonstrated significantly better durable clinical response (OR [95% CrI], 4.89 [1.74–16.0]) and mucosal healing (OR [95% CrI], 9.09 [2.74–40.0]) than placebo (Table 3).

There was no evidence to suggest differences between adalimumab and vedolizumab for durable clinical response and clinical remission (Table 3). Vedolizumab showed significantly improved mucosal healing over adalimumab (OR [95% CrI], 6.72 [1.36–41.0]).

### Results of heterogeneity analysis

The networks did not have any closed loops and were too small to obtain reliable estimates of heterogeneity or to use meta-regression techniques to investigate possible modifiers of treatment effect. However, as all the treatments were connected to a common comparator (placebo), the different response rates for placebo could be assessed as to how much they may have acted as a ceiling effect on the ORs estimated. The placebo rates were found to be reasonably similar across studies for most networks analysed; however, combining different study designs in the maintenance setting resulted in greater variability in the placebo response rates than in the induction phase. A further check of heterogeneity was conducted where duplicate comparisons existed in the networks. These also did not show any significant differences.

## Discussion

Eight RCTs were included in this systematic review of biologics for the treatment of UC in adults, none of which were head-to-head comparisons. Indirect comparisons can provide exploratory insights into the relative efficacy and safety of biologics when no head-to-head studies have been conducted. This study indirectly compared vedolizumab with infliximab, adalimumab and golimumab, assessing the anti-TNF therapy-naïve and anti-TNF therapy-experienced/failure populations separately in recognition of the clinical differences between them. This study adds to the current understanding of the comparative efficacy and safety of biological therapies for UC, encompassing new outcomes and populations.

In the induction treatment of anti-TNF therapy-naïve patients with UC, infliximab demonstrated a significant improvement over adalimumab in clinical response, clinical remission, and mucosal healing. However, there was no evidence to suggest differences between infliximab and vedolizumab, between infliximab and golimumab, or between labelled doses of the other licensed treatments (vedolizumab, adalimumab, and golimumab) for clinical response, clinical remission, or mucosal healing. Credible intervals were typically larger for vedolizumab compared with the other interventions owing to the relatively small placebo sample for the vedolizumab trial, and only one study investigated vedolizumab compared with two studies for infliximab and three studies for adalimumab.

The maintenance studies used different trial designs. Thus, in those studies that did not rerandomise patients at the end of induction, a comparison was only possible using the number of responders at end of induction as a proxy for the number of responders rerandomised at end of induction. Using this method, vedolizumab, in the maintenance treatment of anti-TNF therapy-naïve patients with UC, was significantly better than golimumab, adalimumab, and infliximab for durable clinical response and significantly better than adalimumab for mucosal healing, although CrIs were wide.

The results for the anti-TNF therapy-naïve population are consistent with previous reports [15, 16]. The methods, data sources, study selection and biologics of interest in the review by Danese and colleagues [15] were similar to our study. However, Danese and colleagues [15] included only the anti-TNF therapy-naïve population. Both studies included the same eight RCTs in their MTC. In the Danese and colleagues’ study [15], all biological agents (adalimumab, golimumab, infliximab, and vedolizumab) demonstrated superiority over placebo for induction of clinical response, clinical remission, and mucosal healing, except adalimumab for clinical remission. Results from our analysis were similar, except that in this analysis adalimumab also demonstrated superiority over placebo for clinical remission at end of induction; the results from Danese and colleagues [15] were close to significance. When comparing treatments with each other in the induction setting, Danese and colleagues [15] found that the results of their Bayesian network meta-analyses (NMAs) suggested that infliximab was more likely to induce a favourable clinical outcome than adalimumab. None of the other indirect treatment comparisons reached statistical significance. Our analysis did not find evidence to suggest differences between labelled doses of licensed treatments for clinical response, clinical remission or mucosal healing. It is highly likely that the clinical studies in question were only powered to test to look for a difference with placebo, rather than to test the effect of different doses.

In the maintenance analysis by Danese and colleagues [15], two distinct groups of maintenance studies were formed: group A, with adalimumab and infliximab studies; and group B, with golimumab and vedolizumab studies. The authors did not compare treatments with each other in the maintenance setting. As described in the Methods section, we adjusted for different study designs (including randomisation methods) to allow the inclusion of all of the maintenance studies in our analysis. Mei and colleagues [17] recently conducted an NMA that included an assessment of all the biologics reviewed in the present study at the maintenance time point. However, only results for the entire population were reported; no analyses on anti-TNF therapy-naïve and anti-TNF therapy-experienced/failure subpopulations were conducted. Although the authors reported no significant differences between biologics in terms of clinical remission or mucosal healing [17], a direct comparison against the results from the current study is difficult given the different populations that were analysed.

A similar NMA to the present study was conducted by Stidham and colleagues [16], although only relative risk values were reported. For the anti-TNF–naïve patients in the induction phase, rank order of treatments from best to worst was the same as the present study (i.e., infliximab, golimumab, adalimumab, and then placebo). Vedolizumab was not included in this NMA. All treatments were found to be significantly better than placebo; otherwise, there were no significant differences. The present study found the difference between infliximab and adalimumab to be significant. The difference was probably due to the addition of the Suzuki and colleagues [49] data in the present study, which were not included in the NMA conducted by Stidham and colleagues [16]. A very similar pattern was shown for remission. It was more difficult to make a comparison with the maintenance results presented by Stidham and colleagues [16]. In the studies included by Stidham and colleagues [16], the same patients were followed through both induction and maintenance, whereas the present study also included studies that rerandomised only those patients who had responded after the induction phase. The results from Stidham and colleagues [16] showed very similar values for response across the biologics with all of them significantly better than placebo. The values for remission were higher for infliximab but did not appear to be significantly different to other biologics.

There are limited clinical trial data available on anti-TNF therapy-experienced populations. Data on the group of patients with prior anti-TNF therapy failure were only available for vedolizumab. The only comparator with any similar data was adalimumab, and the data available were for the anti-TNF therapy-experienced population (who by definition may have responded to prior anti-TNF therapy). It is likely that the anti-TNF therapy-failure population is more difficult to treat than the anti-TNF therapy-experienced population, so conclusions from these analyses should be made with caution. The lack of comparable data means it is unclear whether the different mechanism of action of vedolizumab results in improved efficacy in patients who have failed a prior anti-TNF therapy and thus may be less likely to respond to another anti-TNF therapy. Although there were no significant differences in induction or maintenance outcomes of response and remission between vedolizumab and adalimumab, vedolizumab demonstrated significantly improved rates of mucosal healing compared with adalimumab. None of the identified NMAs previously discussed included an analysis of the subpopulation of patients with UC with prior anti-TNF therapy failure and/or experience. However, a comparison was only possible for vedolizumab and adalimumab. Only vedolizumab showed a significant benefit over placebo for response, but no significant difference was found between vedolizumab and adalimumab for either response or remission.

Because of the chronic, recurrent, long-term nature of the condition, patients with UC often require either continuous or intermittent treatment throughout the course of their disease [6]. Thus maintenance results are particularly important. Vedolizumab demonstrated significantly improved rates of mucosal healing compared with adalimumab in both the anti-TNF therapy-naïve and anti-TNF therapy-experienced subpopulations. This endpoint is important from a physician perspective as an important predictor of long-term and short-term disease outcomes [51]. Mucosal healing is associated with sustained clinical remission and reduces the rates of hospitalisation and surgical resection, as well as the direct and indirect costs [52]. Patients with mucosal healing have also been shown to have a significantly higher health-related quality of life [53].

The systematic literature review and MTCs presented here involved the methods recommended by NICE [54] and used the latest robust statistical techniques. A full systematic review was undertaken to identify all relevant published studies, and all studies appropriate for the meta-analysis were included to reduce the risk of search and selection bias. It is important to note that the literature review was conducted in 2014. However, no new biologics have been licensed in UC since the searches were undertaken, and no new pivotal studies have been published. A recently published systematic review by Moćko and colleagues (2016) [55] included database searches undertaken in February 2016 and did not identify any additional studies compared with this review. Nonetheless, results from these MTCs should not be considered as robust as those from RCTs. A number of assumptions were required to be able to make the comparisons possible. The main assumption was that studies were sufficiently homogeneous not to cause any bias in the observed treatment effects.

Where possible, only robust studies of similar design have been included for the MTCs presented. However, to be able to form a network with the maintenance results, studies with different designs were combined. Two maintenance studies had a study design in which patients were randomised to maintenance therapy based on response criteria following induction therapy (GEMINI 1 and PURSUIT-M). This approach aims to reflect real clinical practice in which a patient would be assessed at the end of induction and subsequent treatment decisions would be made according to response status, thus aligning with recent regulatory guidance on study design involving the separation of induction and maintenance phases [22]. Such a design is not unusual in phase 3 trials and helps to reduce the number of patients needed to demonstrate efficacy in situations where induction efficacy is limited and recruitment may be difficult [56]. In comparison, in ULTRA 2, ACT 1, and Suzuki and colleagues [49], patients were randomised to induction and maintenance regimens at baseline.

It is unclear how the differences in the study design will affect the overall OR for the outcomes included in the MTC. In particular, by combining the studies, we have had to assume that patients who responded at 52 weeks had also responded at end of induction and that there was no carryover effect from the induction period to week 52 for all of the endpoints studied. For analysis of durable clinical response in those studies that did not rerandomise, the number of responders at the end of induction and at the end of maintenance was used as a proxy of durable response in the rerandomised studies. The robustness of the efficacy results mainly depends on whether patients fluctuate between responder and non-responder within the study period. If it is unlikely that patients who have not responded by end of induction become responders at the end of maintenance, then the results will be relatively robust. However, if some patients who are non-responders at the end of induction become responders at the end of maintenance, this may bias the results. This change could potentially bias results in favour of placebo for the non-rerandomised studies, as there is likely to be a larger pool of patients who did not respond to placebo compared with those who responded to the comparator treatment. Furthermore, combining different study designs in the maintenance setting resulted in greater variability in the placebo response rates compared with the induction phase. For example, the placebo response rates for induction for anti-TNF–naïve patients ranged from 26% to 45% at the end of induction and from 18% to 53% in the maintenance setting. The placebo response rates were relatively low for GEMINI 1 at 26% and 27% in the induction and maintenance settings, respectively. It should be noted that in rerandomisation studies such as GEMINI 1, the placebo population in the maintenance phase consists of responders withdrawing from active treatment. Due to prolonged pharmacodynamic effects, the positive response they achieved during induction may persist during the initial period of maintenance treatment. An attempt was made to fit a Bayesian model, using the placebo response rates as a proxy for baseline risk as an additional sensitivity analysis; however, there were insufficient data in the network to obtain convergence, which meant that different chains gave different results and findings were inconclusive.

In some analyses, the number of patients experiencing outcomes was very low, which means results can be affected by small changes. For example, the numbers of patients discontinuing due to AEs was very low, particularly in the short-term induction studies. This low discontinuation rate means that one or two patients experiencing one of these events can lead to significant results; hence results should be interpreted with caution.

Because of the small size of the network, only fixed-effects MTCs were conducted. This approach assumes that the true treatment effect is common in all studies comparing the same treatments. Thus, CrIs may be underestimated, and caution is needed when interpreting any significant differences.

The primary analysis presented here is the subgroup analyses by prior anti-TNF therapy experience. We considered this approach because the patient populations differed between studies and the proportion of patients who were anti-TNF therapy naïve may affect results. Undertaking the subgroup analyses not only ensured that similar patient populations were compared but also reduced the size of the networks analysed.

In addition, it was not possible to contact the investigators of unpublished studies that appeared to be complete; therefore, publication bias cannot be ruled out. To assess the risk of bias within the identified studies, a comprehensive assessment of risk of study bias was conducted in line with NICE guidance [26]. This assessment suggested that all of the studies included were conducted appropriately (Fig 2), limiting the possible bias introduced through the study design.

The limitations stated above mean that there are some differences in study design that should be considered when interpreting the results of these analyses. However, even with these limitations, it is argued that the use of NMA allows a useful synthesis of clinical trial evidence where head-to-head evidence is limited.

## Conclusions

Our meta-analysis suggests that vedolizumab is comparable to current biological therapies (adalimumab, infliximab and golimumab) in the treatment of UC for clinical response and clinical remission at induction. In the anti-TNF–naïve population, infliximab demonstrated a significant improvement over adalimumab for these endpoints in the induction setting; however, there was no evidence to suggest differences between infliximab and vedolizumab, between infliximab and golimumab, or between labelled doses of the other biologics (vedolizumab, adalimumab, and golimumab).

In the maintenance setting, there is a suggestion that vedolizumab demonstrates benefits compared with comparators, irrespective of prior anti-TNF–therapy exposure for both durable clinical response and mucosal healing. Notably, mucosal healing is a key endpoint as an important predictor of long-term and short-term disease outcomes [51]. It is associated with sustained clinical remission, reduced rates of hospitalisation and surgical resection, and direct and indirect costs [52], as well as increased health-related quality of life [53].

A head-to-head study is necessary to definitively demonstrate differences in efficacy between the biological therapies used to treat UC.

## Supporting Information

S1 File PRISMA 2009 Checklist. This document contains a completed PRISMA checklist.
(DOCX)

S2 File MEDLINE Literature Search Strategy. This document contains the MEDLINE literature search strategy that was used to perform the update of the systematic review.
(DOCX)

## Abbreviations

ACT: Active Ulcerative Colitis Trial
AE: adverse event
CrI: credible interval
SF: steroid-free
IBDQ: Inflammatory Bowel Disease Questionnaire
MTC: mixed-treatment comparison
NICE: National Institute for Health and Care Excellence
NMA: network meta-analysis
OR: odds ratio
PRISMA: Preferred Reporting Items for Systematic Reviews and Meta-Analyses
PURSUIT-M: Program of Ulcerative Colitis Research Studies Utilizing an Investigational Treatment–Maintenance
PURSUIT-SC: Program of Ulcerative Colitis Research Studies Utilizing an Investigational Treatment–Subcutaneous
RCT: randomised controlled trial
TNF: tumour necrosis factor
UC: ulcerative colitis
ULTRA: Ulcerative Colitis Long-Term Remission and Maintenance With Adalimumab
