# Supplementary material for: Systematic Review with Network Meta-Analysis: Comparative Efficacy of Biologics in the Treatment of Moderately to Severely Active Ulcerative Colitis
Source: PLoS One. 2016 Oct 24;11(10):e0165435. doi: 10.1371/journal.pone.0165435 (PMC5077077; doi:10.1371/journal.pone.0165435)
Supplement: S2 File — This document contains the MEDLINE literature search strategy that was used to perform the update of the systematic review. (DOCX) [file pone.0165435.s002.docx]

MEDLINE Literature Search Strategy: Ulcerative Colitis, Update of Review (Searches conducted 11 February 2014)

| Line # | Search Terms | Results |
| --- | --- | --- |
| Disease area | | |
| #1 | “Colitis, Ulcerative”[MeSH] OR “ulcerative colitis”[Title/Abstract] | 34,224 |
| Clinical studies and systematic reviews | | |
| #2 | (“Randomized Controlled Trial”[Publication Type] OR “Controlled Clinical Trial”[Publication Type] OR “randomized”[Title/Abstract] OR placebo*[Title/Abstract] OR “Clinical Trials as Topic”[MeSH:NoExp] OR “randomly”[Title/Abstract] OR trial*[Title] OR “Randomized Controlled Trials as Topic”[MeSH] OR randomized controlled trial*[Text Word] OR randomised controlled trial*[Text Word] OR randomized clinical trial*[Text Word] OR randomised clinical trial*[Text Word] OR randomized trial*[Text Word] OR randomised trial*[Text Word] OR “random allocation”[Text Word] OR “double blind method”[Text Word] OR “single blind method”[Text Word] OR ((singl*[Text Word] OR doubl*[Text Word] OR treb*[Text Word] OR tripl*[Text Word]) AND (blind*[Text Word] OR mask*[Text Word])) OR allocated random*[Text Word] OR random assignment*[Text Word] OR open-label trial*[Text Word] OR open-label stud*[Text Word] OR non-blinded stud*[Text Word] OR “Clinical Trial, Phase II”[Publication Type] OR “Clinical Trial, Phase III”[Publication Type] OR “Clinical Trial, Phase IV”[Publication Type] OR “Multicenter Study”[Publication Type] OR “Cohort Studies”[MeSH] OR cohort*[Text Word] OR “longitudinal”[Text Word] OR “Follow-Up Studies”[MeSH] OR evaluation stud*[Text Word] OR “Prospective Studies”[MeSH] OR “randomization”[Text Word] OR “randomisation”[Text Word] OR “Double blind procedure”[Text Word] OR “Single blind procedure”[Text Word]) | 2,625,276 |
| #3 | (systematic AND (“Review” [Publication Type] OR “Review Literature as Topic”[MeSH])) OR “Meta-Analysis” [Publication Type] | 163,703 |
| Comparators | | |
| #4 | vedolizumab OR “vedolizumab” [Supplementary Concept] OR MLN0002 OR Entyvio OR infliximab OR “infliximab” [Supplementary Concept] OR Remicade OR adalimumab OR “adalimumab” [Supplementary Concept] OR Humira OR D2E7 | 10,451 |
| #5 | Golimumab OR “golimumab”[Supplementary Concept] | 318 |
| #6 | (“Colitis, Ulcerative/surgery”[Majr] OR “Colectomy”[MeSH] OR “Proctocolectomy, Restorative”[MeSH] OR “Colonic Pouches”[MeSH] OR “Ileostomy”[MeSH] OR “ileal pouch anal anastomosis”[tiab] OR “ileoanal pouch anal anastomosis”[tiab] OR “ileal pouch-anal anastomosis”[tiab] OR “ileoanal pouch-anal anastomosis”[tiab] OR “IPAA”[tiab] OR “Koch pouch”[tiab] OR “Kock pouch”[tiab] OR “continent ileostomy”[tiab] OR “continent ileostomies”[tiab] OR Brooke ileostom*[tiab] OR Brook ileostom*[tiab] OR “ilcorectal anastomosis”[tiab] OR “ileorectal anastomosis”[tiab]) | 20,375 |
| #7 | (“Cyclosporine/therapeutic use”[Majr] OR Cyclosporin[tiab] OR “Cyclosporine A”[tiab] OR Ciclosporin[tiab] OR “Cyclosporin A”[tiab] OR Neoral[tiab] OR Sandimmune[tiab] OR Sandimmun[tiab] OR Restasis[tiab] OR (Sandimmun[tiab] AND Neoral[tiab]) OR “CyA-NOF”[tiab] OR “CyA NOF”[tiab] OR “CsA-Neoral”[tiab] OR “CsA Neoral”[tiab] OR “CsANeoral”[tiab]) | 30,674 |
| Exclusionary terms | | |
| #8 | “Animals”[MeSH] NOT “Humans”[MeSH] | [3,857,](http://www.ncbi.nlm.nih.gov/pubmed/?cmd=HistorySearch&querykey=12)919 |
| #9 | “Comment”[Publication Type] OR “Editorial”[Publication Type] OR “Letter”[Publication Type] OR “Clinical Trial, Phase I”[Publication Type] OR “Case Reports”[Publication Type] OR “case study”[Text Word] OR “case studies”[Text Word] | [2,840,](http://www.ncbi.nlm.nih.gov/pubmed/?cmd=HistorySearch&querykey=13)559 |
| Total | | |
| #10 Golimumab | (#1 AND (#2 OR #3) AND #5) NOT (#8 OR #9) No limits | 9 |
| #11 Biologics | (#1 AND (#2 OR #3) AND #4) NOT (#8 OR #9) No limits | 405 |
| #12 Surgery/cyc | (#1 AND (#2 OR #3) AND (#6 OR #7)) NOT (#8 OR #9) No limits | 1,538 |
| #13 Biologics | (#1 AND (#2 OR #3) AND #4) NOT (#8 OR #9) Filters: Publication date from 2013/04/01 OR (“2013/04/01”[Date - Entrez]: “3000”[Date - Entrez]) | 61 |
| #14  Biologics including Golimumab | #10 OR #13 | 65 |
| #15 Surgery.cyc | (#1 AND (#2 OR #3) AND (#6 OR #7)) NOT (#8 OR #9) Filters: Publication date from 2013/04/01 OR (“2013/04/01”[Date - Entrez]: “3000”[Date - Entrez]) | 41 |
| #16  Total | #14 OR #15 | 96 |
